# Fabrication of a 3D Multi-Depth Reservoir Micromodel in Borosilicate Glass Using Femtosecond Laser Material Processing

**DOI:** 10.3390/mi11121082

**Published:** 2020-12-06

**Authors:** Ebenezer Owusu-Ansah, Colin Dalton

**Affiliations:** Department of Electrical & Computer Engineering, Schulich School of Engineering, University of Calgary, 2500 University Drive NW, Calgary, AB T2N 1N4, Canada; cdalton@ucalgary.ca

**Keywords:** micromodels, porous media, 3D multi-depth channels, laser machining, femtosecond laser micromachining, femtosecond laser material processing, micro/nanotechnology fabrication

## Abstract

Micromodels are ideal candidates for microfluidic transport investigations, and they have been used for many applications, including oil recovery and carbon dioxide storage. Conventional fabrication methods (e.g., photolithography and chemical etching) are beset with many issues, such as multiple wet processing steps and isotropic etching profiles, making them unsuitable to fabricate complex, multi-depth features. Here, we report a simpler approach, femtosecond laser material processing (FLMP), to fabricate a 3D reservoir micromodel featuring 4 different depths—35, 70, 140, and 280 µm, over a large surface area (20 mm × 15 mm) in a borosilicate glass substrate. The dependence of etch depth on major processing parameters of FLMP, i.e., average laser fluence (${\mathrm{LF}}_{\mathrm{av}}$), and computer numerically controlled (CNC) processing speed (${\mathrm{PS}}_{\mathrm{CNC}}$), was studied. A linear etch depth dependence on ${\mathrm{LF}}_{\mathrm{av}}$ was determined while a three-phase exponential decay dependence was obtained for ${\mathrm{PS}}_{\mathrm{CNC}}$. The accuracy of the method was investigated by using the etch depth dependence on ${\mathrm{PS}}_{\mathrm{CNC}}$ relation as a model to predict input parameters required to machine the micromodel. This study shows the capability and robustness of FLMP to machine 3D multi-depth features that will be essential for the development, control, and fabrication of complex microfluidic geometries.

## 1. Introduction

The use of micromodels, also known as porous media, for microfluidic transport investigations has been extensively studied in the literature for many applications, such as oil recovery [1,2,3,4,5] and carbon dioxide storage [6,7,8,9,10,11] processes. For example, silicon and glass-based micromodels have been used to study pore-scales to understand oil-water-solid interactions, multiphase flow, and the dynamics of microemulsions in enhanced oil recovery processes [4,5,11,12]. This is due to the ability to fabricate micromodels to mimic the three dimensional, multiple depths naturally occurring in porous media (such as oil-bearing rock formations), and the ease to integrate them with optical instruments for real time and in-situ observation of complex flow behaviour [13]. Naturally occurring porous media consist of complex 3D (multiple depth) networks of pores and throats that makes them challenging to study with 2D (uniform depth) micromodels, as the physics of the third dimension, which are critical for understanding flow in porous media, cannot be captured. For example, oil and bubble break-up in multiphase flow is largely dependent on capillary snap-off, a mechanism known to occur when sizes of throats are smaller than pore bodies in the two dimensions that are perpendicular to the flow direction, making it difficult for multiphase flow investigations using 2D micromodels [14,15,16,17]. Therefore, 3D micromodels are essential for studying transport in porous media, including emulsion flow, two phase displacement, three-phase flow, foam flow, etc., that has high dependence on capillary effects. 

The most widely used non-additive manufacturing and conventional method to fabricate 3D micromodels is photolithography, which involves the transfer of a predesigned pattern from a mask to a substrate material, typically glass, followed by a wet chemical etch to define the features in the glass [17]. The process involves several wet processing steps, the need for photomasks, and complicated multi step processes requiring many items of fabrication equipment. Also, the approach suffers from mask undercut due to isotropic etching of substrate by the etchant (typically hydrofluoric acid, HF, for glass) that negatively impacts the ability to control etch feature sizes and resolution, making it difficult to fabricate 3D features with multiple depths in the same substrate [17,18]. Recent progress in wet photolithography includes the work of Xu et al., who fabricated a two-depth 3D micromodel in the same glass substrate by varying the depth difference between the pore body and throat [16]. Also, Yun et al. used a similar approach to achieve two depths in silicon by repeating the etch process twice [19]. These are time consuming processes, requiring multiple masks, and provide little to no control on lateral separation between etched features. The fabrication of micromodels using dry etching photolithographic methods, where the photoresist or masking material is exposed to a plasma of reactive gasses such as Cl_2_, O_2_, and BCl_3_, to remove the unprotected substrate material, has been reported [17,20,21,22]. In comparison to wet etching, dry etching methods, such as reactive ion etching (RIE), allows for control on the etch direction that results in vertical channel sidewalls; however, RIE requires sophisticated facilities [17] and is also limited to the fabrication of 2D channels (i.e., a single uniform depth throughout) [21,22]. On the other hand, additive manufacturing methods, e.g., stereolithography or 3D printing, can be used to make 3D micromodels from many materials, including resins, polymers, and hydrogels; however, they are limited to larger than micron sized features due to the spatial requirements for solidification of the liquid materials and are typically not optically transparent [17,23,24,25]. 

Femtosecond (fs) laser material processing (FLMP) is a simpler approach that has been used to date to machine 2D microchannel features into optically transparent materials such as borosilicate glass [26]. Others have used FLMP together with wet etch processes to produce 2D structures in photosensitive glass substrates [27]. Here in this study, we showed the capability of using FLMP with no additional wet etch methods to fabricate 3D microstructures consisting of 4 different depths in the same borosilicate glass substrate for use as a reservoir micromodel. Details of the FLMP method and its advantages over widely used conventional micro/nanotechnology (MNT) fabrication approaches are given in the next section. 

### Femtosecond Laser Material Processing (FLMP)

FLMP technique allows the development, control, and fabrication of MNT systems such as microfluidic and lab-on-a-chip devices that are not easily accomplished with traditional methods, such as photolithography [28,29,30]. Unlike conventional MNT fabrication, in FLMP, there is no need for photomasks or multiple coating and chemical etching procedures [28]. FLMP involves a computer numerically controlled (CNC) motion that can machine complex patterns through cycles of focused laser beam passes with high precision. Applications of FLMP includes the fabrication of microfluidic devices such as micro- [31,32], hydro-dynamic fluid pumps [29,33], and dielectrophoretic assays [30,34]. Also, FLMP has allowed internal machining of quartz to create waveguides [35,36,37] in optical systems, and the fabrication of complex X-ray masks in thin sheets of tungsten, a material that is not suited for chemical based etch methods due to its non-uniform structure that leads to uneven etch profiles [38]. 

When a femtosecond (fs) laser pulse incidents on a material, photon absorption occurs on a timescale (~10^−14^–10^−13^ s) that is shorter than the electron-phonon coupling relaxation process (~10^−12^–10^−11^ s), delivering energy to the electrons while leaving the ions and the lattice “cold”. This ensures that within the duration of the fs pulse, there is little to no thermal energy transfer to the lattice which decouples the optical absorption processes from lattice thermalization processes. The energy absorbed by the electrons causes excitation which breaks the bonds formed by these electrons with minimal heating of the material substrate [39]. In comparison to long laser pulses (e.g., nanosecond) [40,41,42], fs laser pulses produce high peak electric fields (~10^12^ V/m) which are approximately 3 orders of magnitude greater than the electric field (10^9^ V/m) that binds electrons to atoms [39,43]. This make fs laser processing versatile to process a wide variety of materials including optically opaque and transparent materials, such as metals, glass, and silicon wafers. The high peak electric field allows non-linear optical absorption processes, such as multiphoton absorption and tunneling ionization [44], within the material substrate when the laser beam is tightly focused, resulting in bond breakage and the ablation of material from the exposed surface. The wavelength of most fs lasers used for FLMP of wide bandgap materials (e.g., semiconductors and glass) is typically > 750 nm (1.65 eV), making the process nearly wavelength independent as the bandgap of these materials are mostly higher (~>2 eV) than the photon energy [45]. When the energy of an incident photon is larger than the bandgap of the material substrate, absorption occurs, and electrons are excited to the conduction band. On the contrary, optical absorption by electrons does not occur when the photon energy is smaller than the bandgap. However, when light with large peak electric fields, such as those generated by fs laser beams, are focused to produce an extremely high density of photons, electron absorption is possible through multiple photon absorption at several virtual states. This multiphoton absorption process allows electron excitation into the conduction band. For ablation to occur, the density of free electrons in the conduction band should reach a critical density that is achieved beyond a threshold laser fluence (optical breakdown) which is material dependent [46].

The FLMP technique is ideally suited to micro-structuring, as the ultra-short pulse width of the fs laser is shorter than the thermal diffusion times of most materials, including metals, ceramics, and glass [47,48,49]. In FLMP, the formation of a heat affected zone (HAZ), when a large portion of a laser pulse’s energy is transformed to heat around the irradiated area and causes material damage, is significantly suppressed. Thus, with FLMP, there is little or no HAZ around the exposed site, resulting in less damage to the substrate material than conventional CO_2_, nanosecond, and long pulse lasers. This allows for fine control of feature sizes not possible with CO_2_ and long pulse lasers, enabling the fabrication of high-precision and high-quality MNT devices [28,39,44,50,51]. Also, an additional feature of the FLMP technique is the ability to easily make changes to a design by modifying the machining pattern on a computer, i.e., editing a CAD file. This significantly reduces the cost of prototyping by removing the need for multiple high-resolution photomasks and allows for a fast-iterative design cycle. 

Recent investigations in FLMP have included efforts on how to effectively control the processing parameters, such as CNC speed, fluence (energy density), focused laser beam size, wavelength, and repetition rate [47,52,53,54,55,56,57]. These processing parameters have a significant effect on the properties of the resultant material etch parameters, such as etch profile (including cleanliness of the cut-edge), depth, feature size resolution, and surface roughness. Kam et al. used FLMP to machine multi-depth microchannel networks onto a silicon substrate for use as a gas exchanger [52]. It was found that the processing speed had a significant effect on the surface quality and the processing time. Hayden studied a simple 3D computer simulation tool to help predict some of the resultant etch parameters of FLMP on sodalime glass, borosilicate glass, and silicon substrates [47]. These investigations are important to harness any latent potential of the FLMP technique. Here, a study on the effect of average laser fluence (${\mathrm{LF}}_{\mathrm{av}}$), and CNC processing speed (${\mathrm{PS}}_{\mathrm{CNC}}$) to determine their relationship with the resultant etch depth in a borosilicate glass substrate is presented. The obtained relations were then used as models to guide the fabrication of 3D multi-depth features into a borosilicate glass substrate with 4 different depths for use as a reservoir micromodel. 

## 2. Materials and Methods

**Figure 1:**
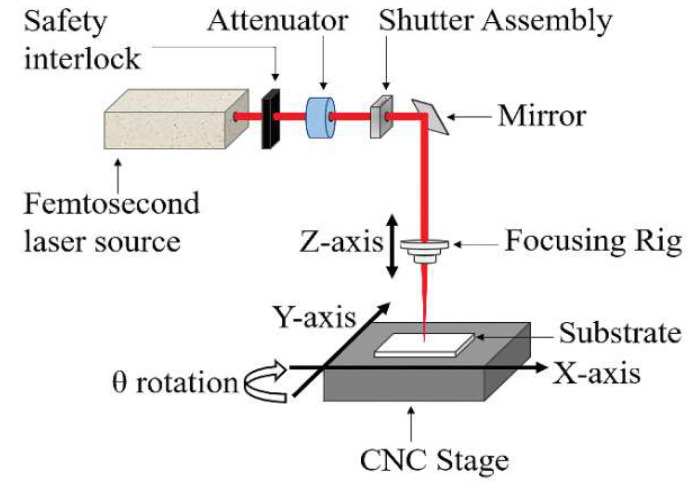
Schematic illustration of the femtosecond laser material processing (FLMP) setup. The path of the laser beam is fixed while the CNC stage allows XYZθ motions.

A detailed description of the FLMP workstation setup used for this study has previously been reported [28,47]. However, for convenience and minor changes in the optical path, a brief description is given here. A schematic representation of the FLMP workstation is shown in Figure 1. It consists of a Ti:Sapphire Regenerative Amplifier Laser System (Spectra-Physics, Spitfire Pro, USA) that produces 800 nm infra-red (IR) radiation with 100 femtosecond (fs) pulse duration. The maximum output laser power arriving at the working piece substrate was 2.5 W when measured with a power meter (Ophir Meter) at a repetition rate of 1 kHz. The wavelength was tunable from 780–820 nm, while the repetition rate could be varied from 0.1–1 kHz. A summary of the input processing parameters used in this study are given in Table 1. The laser system was synchronized to a CNC stage (Aerotech, Inc., Pittsburgh, PA, USA) that allows XYZθ motions. Precise motion control, positioning, and machining were possible over a large area of 150 mm × 150 mm.

The pattern to be machined was first designed using CAD/CAM software (Alphacam 2019 R1) and converted to a G-code text file, which was uploaded onto a computer that controls the motion stages. The path of the CNC motion stage enables the laser beam, when on, to create the desired pattern in the substrate located on the workpiece. This allows programmable, accurate, and repeatable motions for patterning complex MNT features. The laser beam path, which is fixed, was directed through a set of optical components, including safety interlock, attenuator, opto-mechanical shutter assembly, mirrors, and a focusing lens (housed in a focus rig) onto the CNC motion stage. The vertical *Z* axis motion allows the laser beam to be focused on different thicknesses of material substrates with the aid of an alignment camera and light mounted above the focus rig. The borosilicate glass substrate was held in place on the CNC stage by a vacuum suction source. Material properties of the borosilicate glass (McMaster-Carr^®^, Part # B84760365) were; density: 2440 kg/m^3^, hardness: Knoop 418 KHN100, refractive index: 1.47, and the two largest components by % composition were SO_2_: 70–87% and BO_3_: 1–20%. 

The attenuator was used to control the amount of laser energy arriving at the material substrate. During FLMP, the laser beam was always on, and therefore, the shutter assembly was needed to block off the beam when no machining was required, especially when the CNC stage was moving to a new location to machine a new feature on the substrate. The overhead camera and light were used for alignment purposes. An exhaust was mounted near the laser beam-substrate surface to remove machined debris during all FLMP experiments. To investigate etch depth dependence on ${\mathrm{LF}}_{\mathrm{av}}$, and ${\mathrm{PS}}_{\mathrm{CNC}}$, square features (1500 µm × 1500 µm) were machined in borosilicate glass substrates where all FLMP parameters were held constant while varying ${\mathrm{LF}}_{\mathrm{av}}$ and ${\mathrm{PS}}_{\mathrm{CNC}}$, respectively. To machine features larger than the focused laser beam size, toolpaths consisting of several lines were generated for each feature. The pitch, spacing between the toolpath lines (center-to-center), was experimentally determined as it affects the machining time and the roughness of the etched surface. The extensive data on the effect of pitch variations on material substrate roughness will be covered in another manuscript. A 5 µm pitch and single laser beam pass were used for this work unless stated otherwise. After laser machining, the borosilicate glass substrates were immersed in an isopropyl alcohol sonication bath for 30 mins to remove remaining debris before a contact surface profilometer (P-6, KLA Tencor) with a 2 µm tip was used to characterize the etch profiles. Optical microscopy images were taken with Mitutoyo (Ultraplan FS110) while regression analysis data fitting was performed using OriginPro^®^ software (version 8).

## 3. Results and Discussion 

### 3.1. FLMP Etch Profiles 

**Figure 2:**
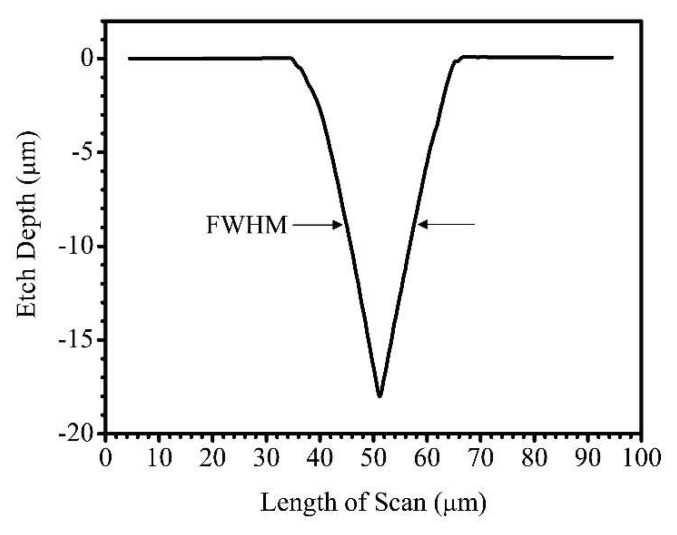
Line profile scan across 4 mm-length line feature machined into borosilicate glass using FLMP at ${\mathrm{PS}}_{\mathrm{CNC}}$ of 0.25 mm/s and 0.617 mJ power. The profile was recorded at 2 µm/s, 10 Hz, and 2 mg applied force. The full-width at half-maximum (FWHM) value of 12.3 µm of the Gaussian-like etched profile was determined as the spot size of the focused laser beam diameter.

The size of the focused laser beam spot was experimentally determined by systematically varying the vertical Z position of the focus lens (*f* = 25 mm, F/0.6, Edmund Optics^®^) to machine 4 mm-length line features on the borosilicate glass substrate. At ${\mathrm{PS}}_{\mathrm{CNC}}$ of 0.25 mm/s and 0.617 mJ power, an expected Gaussian-like etch profile was produced, as shown in the 2D line profile scan in Figure 2. For Gaussian-like profiles, the full-width at half-maximum (FWHM) value of 12.3 µm was determined as the diameter of the focused laser beam [58]. This was used to calculate the circular area in the determination of all ${\mathrm{LF}}_{\mathrm{av}}$ values reported in this work.

**Figure 3:**
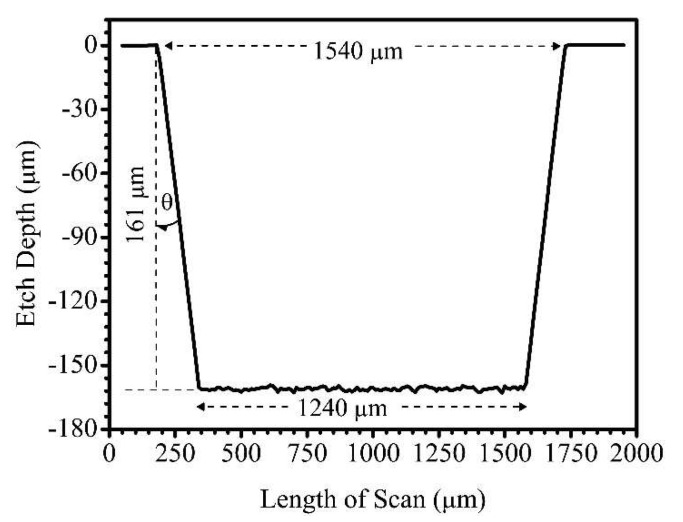
Line profile scan of FLMP etch area (1500 µm × 1500 µm) machined into borosilicate glass substrate at ${\mathrm{PS}}_{\mathrm{CNC}}$ of 0.1 mm/s and ${\mathrm{LF}}_{\mathrm{av}}$ of 329.06 J/cm^2^. The profile was recorded at 5 µm/s, 10 Hz, and 2 mg applied force. The inclined etch surfaces make a contact angle (θ) of ~8° with the vertical plane.

The profile of an etched area covering 1500 µm × 1500 µm was also etched at a pitch of 5 µm, ${\mathrm{PS}}_{\mathrm{CNC}}$ of 0.1 mm/s, and ${\mathrm{LF}}_{\mathrm{av}}$ of 329.06 J/cm^2^. Figure 3 is a line profile scan across the etched area. The profile shows two inclined lines that reveal a symmetrical (isosceles) trapezoid geometry in comparison to the vertical lines of a rectangle. This was a direct consequence of the Gaussian-like profile of the focused laser beam as shown in Figure 2. The difference between the programmed G-code width of 1500 µm and the resultant machined width of ~1540 µm represents an offset value of ~40 µm that could be accounted for during subsequent CAD/CAM designs. However, no offset in width was factored into the designs reported in this work since that was not the focus of the study. The analysis of the trapezoid geometry also showed that the inclined etch surfaces make ~8° contact angle with the vertical plane. 

### 3.2. Etch Depth Dependence on Average Laser Fluence (${LF}_{av}$)

**Figure 4:**
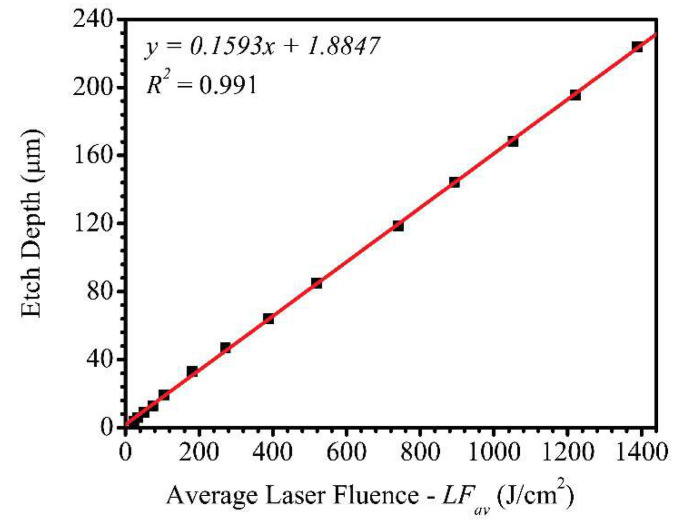
A plot showing the linear dependence of etch depth on average laser fluence (${\mathrm{LF}}_{\mathrm{av}}$) while keeping all processing parameters constant, such as ${\mathrm{PS}}_{\mathrm{CNC}}$ at 0.25 mm/s. Legend—experimental data points: black squares, data fitting: red trace.

The dependence of etch depth on ${\mathrm{LF}}_{\mathrm{av}}$ was studied by varying ${\mathrm{LF}}_{\mathrm{av}}$ while keeping all other parameters constant. The average laser power was varied from 0.017–1.65 W, which corresponds to ${\mathrm{LF}}_{\mathrm{av}}$ values of 14.31–1388.62 J/cm^2^, respectively. A linear expression

y = 0.1593x + 1.8847(1)

with an excellent $R^{2}$ value of 0.991 was obtained as shown in Figure 4. This shows that the etch depth has a strong linear dependence on ${\mathrm{LF}}_{\mathrm{av}}$.

The minimum threshold average laser fluence (${\mathrm{LF}}_{\mathrm{av}}^{\mathrm{th}}$) required to etch the borosilicate glass substrate was also investigated. Below 22.72 J/cm^2^ (27 µJ), it was found that there was no laser etch on the borosilicate glass substrate at ${\mathrm{PS}}_{\mathrm{CNC}}$ of 0.25 mm/s. The ${\mathrm{PS}}_{\mathrm{CNC}}$ was further reduced systematically down to 0.025 mm/s, but no laser etch features were observed. Therefore, 22.72 J/cm^2^ was determined as the ${\mathrm{LF}}_{\mathrm{av}}^{\mathrm{th}}$ required for a successful FLMP on borosilicate glass. It must be mentioned that a borosilicate glass with different material composition and specification would have a different ${\mathrm{LF}}_{\mathrm{av}}^{\mathrm{th}}$. The corresponding depth at the determined ${\mathrm{LF}}_{\mathrm{av}}^{\mathrm{th}}$ was 3.9 µm, and this implied that the y-intercept value of 1.8847 µm at 0 J/cm^2^ had no physical meaning. This was because the minimum etch depth that could be achieved was 3.87 µm at ${\mathrm{LF}}_{\mathrm{av}}^{\mathrm{th}}$ = 22.72 J/cm^2^, therefore, one could only expect an etch depth of 1.885 µm if the ${\mathrm{LF}}_{\mathrm{av}}^{\mathrm{th}}$ value was less than 22.72 J/cm^2^. Hence, the obtained linear relation was applicable to predict etch depths that were ~≥4 µm deep. 

In comparison to the literature, a report by Shin et al. [54] who used FLMP to machine a PDMS substrate obtained a linear relation. The group used 190 fs laser system that produced 343 nm wavelength UV radiation with a maximum average power and pulse energy of 1.8 W and 375 µJ, respectively, at 600 kHz repetition rate. They used a focused laser beam diameter of 5 µm and a relatively fast ${\mathrm{PS}}_{\mathrm{CNC}}$ of 500 mm/s, and explored etch depth dependence on increasing ${\mathrm{LF}}_{\mathrm{av}}$ in the range of 19.11–382.16 J/cm^2^ and obtained a linear relationship. In addition, they studied etch depth dependence on the number of laser beam passes (5–15 multiple passes) on the same surface and observed a similar linear relationship. A similar observation was made by Kam et al. [52] for a silicon wafer substrate. In their study, a 1040 nm wavelength laser with a ~600 fs pulse duration that produced a maximum output power of ~2 W at 200 kHz with a beam spot size of 22 µm diameter was used to machine silicon wafer substrates at 20 µm pitch followed by wet chemical methods. They kept the fluence constant at 3.09 J/cm^2^ (9.72 µJ) and increased the number of laser beam passes (multiple pass) as was used by Shin et al. [54]. At constant ${\mathrm{PS}}_{\mathrm{CNC}}$ of 30, 120, 480, and 1920 mm/s, a linear relationship for etch depth dependence on multiple number of laser beam passes from ~1–157 was obtained. Though the multiple pass approach is slightly different from increasing the ${\mathrm{LF}}_{\mathrm{av}}$ as used in our study, the previous work by Shin et al. [54] has shown that the two methods are comparable as they produce linear relationships for the etch depth dependence. 

Also, Crawford et al. investigated etch depth dependence on ${\mathrm{LF}}_{\mathrm{av}}$, and ${\mathrm{PS}}_{\mathrm{CNC}}$ by machining linear grooves in a silicon substrate using 800 nm wavelength laser with 150 fs pulse duration [59]. At ${\mathrm{PS}}_{\mathrm{CNC}}$ of 0.1–0.5 mm/s, multiple linear relations of etch depths at different ${\mathrm{LF}}_{\mathrm{av}}$ regimes were observed. At a lower ${\mathrm{LF}}_{\mathrm{av}}$ regime (~< 1.1 J/cm^2^), a linear etch depth dependence on fluence was obtained with a slow rise gradient, while at relatively higher ${\mathrm{LF}}_{\mathrm{av}}$ regime (~1.1–10 J/cm^2^) another linear relationship with a sharp rise gradient was obtained. In comparison to our work on borosilicate glass substrate, the ${\mathrm{LF}}_{\mathrm{av}}^{\mathrm{th}}$ value of 22.72 J/cm^2^ required to observe any etch feature on the substrate was already higher than the highest ${\mathrm{LF}}_{\mathrm{av}}$ (10 J/cm^2^) investigated by Crawford et al. to etch silicon substrates [59]. However, a similar work by Lee et al. who used 775 nm laser radiation with 150 fs pulse duration to machine silicon wafers over a relatively wide ${\mathrm{LF}}_{\mathrm{av}}$ range (<1000 J/cm^2^) also obtained two linear relations for etch depth as a function of ${\mathrm{LF}}_{\mathrm{av}}.$ At low (<10 J/cm^2^) and high (10–1000 J/cm^2^) ${\mathrm{LF}}_{\mathrm{av}}$ regimes, linear logarithmic relationships with slow and fast rise gradients were observed, respectively [48]. These FLMP literature reports, especially the works of Crawford et al. and Lee et al. on silicon wafer substrates strongly support the fact that there are multiple linear etch depth relations, one at low fluence and the other at high fluence. Our study has shown that there is a single etch depth linear dependence on ${\mathrm{LF}}_{\mathrm{av}}$ when using FMLP to machine a borosilicate glass substrate. This, to the best of our knowledge, is the first-time experimental determination of such a relation for a borosilicate glass substrate. This is important for future MNT fabrication involving borosilicate glass substrates, such as reservoir micromodels, due to the excellent mechanical strength, exceptional optical transparency, high chemical resistance, and high thermal resistance to the rapid temperature variations of borosilcate glass [60]. 

### 3.3. Etch Depth Dependence on CNC Processing Speed (${\mathrm{PS}}_{\mathrm{CNC}}$)

**Figure 5:**
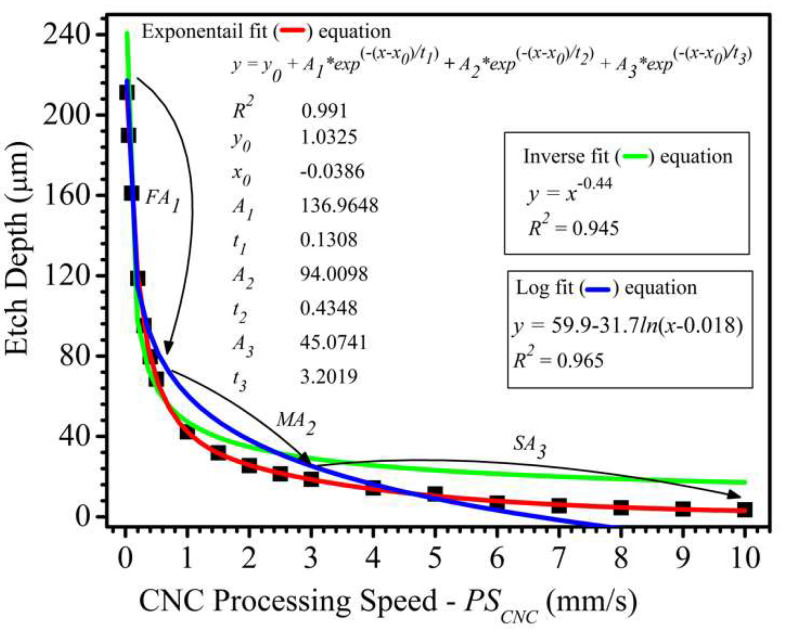
A plot showing an inverse (green trace), logarithmic (blue trace) and a three-phase exponential decay (red trace) dependence of etch depth on CNC processing speed (${\mathrm{PS}}_{\mathrm{CNC}}$). All processing parameters were kept constant, including average laser fluence (${\mathrm{LF}}_{\mathrm{av}}$) at 329.06 J/cm^2^. Approximate portions of the plot that shows fast, medium, and slow exponential decays are represented by ${\mathrm{FA}}_{1}$, ${\mathrm{MA}}_{2}$, and ${\mathrm{SA}}_{3}$, with pre-exponential decay factors of 136.9648, 94.0098, and 45.0741 µm, respectively. Legend—experimental data points: black squares, inverse data fitting: green trace, logarithmic data fitting: blue trace, exponential data fitting: red trace.

The dependence of etch depth on ${\mathrm{PS}}_{\mathrm{CNC}}$ was studied by varying the ${\mathrm{PS}}_{\mathrm{CNC}}$ from 0.025–10 mm/s while keeping all other parameters constant, including ${\mathrm{LF}}_{\mathrm{av}}$ of 329.06 J/cm^2^. All borosilicate glass substrates used in this study were from the same batch unless mentioned otherwise. The data set was fitted to inverse (green trace), logarithm (blue trace), and exponential (red trace) relations as shown in the graph in Figure 5. The fitting results showed that our data agrees more with the exponential plot than the inverse and logarithm relations. This is supported by a better $R^{2}$ value of 0.991 for the exponential fitting relative to 0.945 and 0.965 for the inverse and logarithm fitting, respectively. The inverse relation was found to be the worst fitting plot to the experimental data. Here, we obtained a three-phase exponential decay dependence of etch depth on ${\mathrm{PS}}_{\mathrm{CNC}}$. It is observed that the deviation of the inverse fitting curve from the data points increases at ${\mathrm{PS}}_{\mathrm{CNC}}$ > 1 mm/s. 

Some literature reports of FLMP on silicon wafer substrates have reported that the etch depth has an inverse dependence on the ${\mathrm{PS}}_{\mathrm{CNC}}$. In the previously discussed work by Crawford et al., an inversely proportional relationship for etch depth (<25 µm) as a function of ${\mathrm{PS}}_{\mathrm{CNC}}$ (0.05–1 mm/s) was obtained when an 800 nm wavelength laser with 150 fs pulse duration was used to machine silicon substrates [59]. A similar observation was made by Kam et al., who explored etch depth (<250 µm) dependence on ${\mathrm{PS}}_{\mathrm{CNC}}$ (0.1–1.9 mm/s) by using a 1040 nm wavelength laser with ~600 fs pulse duration to machine silicon wafer substrates [52]. The work of these groups corroborates an earlier work by Ameer-Beg et al., who used a 790 nm wavelength laser with ~170 fs pulse duration to machine fused silica substrate [61]. Ameer-Beg et al. obtained an inversely proportional dependence for etch depth (<40 µm) on ${\mathrm{PS}}_{\mathrm{CNC}}$ (1–7 mm/s). It is important to note that most of these literature works found their experimental data obtained for silicon wafer to be in good agreement with an inverse relation, while in our study for borosilicate glass, the inverse relation was the worst to agree with the data.

Also, in the FLMP work of Lee et al. [48], also on silicon wafer substrates that was discussed in Section 3.2, they explored etch depth (<6 µm) dependence on ${\mathrm{PS}}_{\mathrm{CNC}}$ (0.5–2.5 mm/s) at a relatively lower ${\mathrm{LF}}_{\mathrm{av}}$ range (1.56–6.26 J/cm^2^) in comparison to the value of 329.06 J/cm^2^ used in this work. They found that etch depth has a one-phase exponential decay dependence on ${\mathrm{PS}}_{\mathrm{CNC}}$. Unlike silicon wafer substrates, there are no such literature studies on borosilicate glass. This is largely due to the widespread use of silicon wafers for MNT fabrication. As previously mentioned in Section 3.2, borosilicate glass is an excellent material for use as reservoir micromodels and microfluidic devices due to its unique material properties such as high optical transparency and high resistance to rapid thermal changes [60]. Hence, it will be important to the MNT community to know borosilicate’s fundamental laser-material interaction relationships, such as the etch depth dependence on processing speed. Here, we report the observation of a three-phase exponential decay dependence of etch depth on ${\mathrm{PS}}_{\mathrm{CNC}}$ for a borosilicate glass substrate. From regression analysis data fitting, values of 136.965, 94.010, and 45.074 µm were obtained which corresponds to the pre-exponential decay factors for the fast, medium, and slow decay regions, respectively. As shown in Figure 5, there is a good statistical agreement between the experimental data points (black squares) and the three-phase exponential fit (red trace) with an excellent $R^{2}$ value of 0.991. 

It is worth mentioning that the range of etch depths (3.47–223.8 µm), ${\mathrm{LF}}_{\mathrm{av}}$ (14.31–1388.62) and ${\mathrm{PS}}_{\mathrm{CNC}}$ (0.025–10 mm/s) investigated in this work is wider than those reported in the literature for commonly used substrates, such as silicon and silica [48,52,59,61]. Pfeiffer et al. have reported on the FLMP of tungsten carbide and steel substrates using 775 nm wavelength radiation with 150 fs pulse duration [53]. Part of their studies explored etch depth dependence on ${\mathrm{LF}}_{\mathrm{av}}$ over a total depth range <220 µm, and ${\mathrm{LF}}_{\mathrm{av}}$ of 0.2–11 J/cm^2^ for the materials. In other studies, polymer substrates such as poly(methyl methacrylate) (PMMA) have been machined with FLMP over etch depth, ${\mathrm{LF}}_{\mathrm{av}}$, and ${\mathrm{PS}}_{\mathrm{CNC}}$ of <130 µm, 0.11–1.72 J/cm^2^, and 0.5–10 mm/s, respectively [62]. Hence, the wide range of FLMP parameters explored in this study (Table 1) would be applicable and useful as a guide to future FLMP investigations involving many material substrates, including glass, metals, composite materials (e.g., tungsten carbide), and polymers. 

### 3.4. Fabrication of 4 Depth 3D Reservoir Micromodel

**Figure 6:**
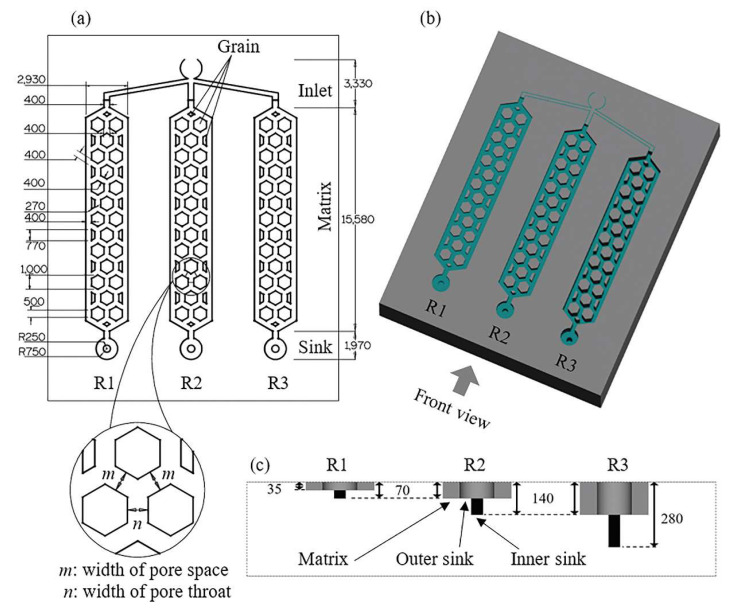
CAD schematic illustration of the 3D multi-depth reservoir (R) micromodel. (**a**) a 2D top view showing a description of all components of the micromodel and their dimensions in µm units, and a zoom-in portion that shows the pore body bounded by 3 solid hexagon grains, and the uniform widths of the pore space (m) and pore throat (n) which gives an aspect ratio (mn) = 1. (**b**) a 3D top view design showing the borosilicate glass substrate (grey) and the etch area (green) with multiple depths. (**c**) a front view of (**b**), grey arrow direction, showing the 4 depths of the reservoir micromodel –35, 70, 140, and 280 µm relative to the surface of the borosilicate substrate. The notations used here are R1 matrix, R1 outer sink, and R1 inner sink that represents the main reservoir matrix, the large outer circular sink, and the small inner circular sink of reservoir 1, etc. Emphasis was placed on the reservoir matrices and sinks, therefore portions (grey area) of the inlet channel was not etched as shown in (**b**).

A CAD representation of the reservoir micromodel made in Alphacam is shown in Figure 6. The 2D (Figure 6a) and 3D (Figure 6b) top view designs show 3 porous reservoirs (R1, R2, and R3) with inlet channels connected to a common sink. The reservoirs have the same XY dimensions (Figure 6a) but different Z dimensions (depths) as shown in the 3D top view (Figure 6b) and the front view, Figure 6c, that details the various etch depths relative to the substrate surface. The following notations were used; R1 matrix, R1 outer sink, and R1 inner sink that represents the main reservoir matrix, the large outer circular sink, and the small inner circular sink of reservoir 1, etc. Another notation used here is layer 1 and layer 2 which represents the matrices/outer sinks and inner sinks of the reservoirs, respectively. The circular sinks at the bottom of the reservoir matrices have two depths—the large outer circular features have the same depths as their respective R matrices, while the smaller inner circular features have twice as much depth as their respective R matrix depth (Figure 6c). This CAD model shows a total of 4 different depths, i.e., 35 µm (R1), 70 µm (R1 and R2), 140 µm (R2 and R3), and 280 µm (R3) in the same substrate. It is worth mentioning that 6 or more multiple etch depths could have been achieved by using different etch depths for the matrix, outer and inner sinks of each reservoir. However, common etch depths such as 70 µm and 140 µm were used in R1/R2 and R2/R3, respectively, to determine whether etch depths were repeatable among reservoirs which were machined at different times. 

Pore dimensions, such as size and shape, are known to influence fluid flow in porous media [9,10,63,64,65]. Here, the pore body is bounded by 3 solid hexagon grains as shown in the zoom-in inset of Figure 6a. The pore space, *m*, longest distance between two solid grains, and pore throat, *n*, shortest distance between two solid grains [9], have uniform width of 400 µm, producing an aspect ratio mn=1. The solid grains in the reservoir matrix are mainly composed of large hexagons and small trapezoid geometries. The dimensions of the hexagons were 1000 µm (length) and 800 µm (breath) which gives an aspect ratio of 1.25. Similarly, the length and breadth of the trapezoid grains were 650 and 200 µm, respectively, producing an aspect ratio of 3.25. Each reservoir had a total surface area and etch surface area of 6.43 × 10^7^ and 4.99 × 10^7^ µm^2^, respectively, which results in a surface porosity of 77.6%. The different depths of R1, R2 and R3 produced total etch volumes of 2.25 × 10^9^, 4.82 × 10^9^, and 9.00 × 10^9^ µm^3^, respectively. Prior to machining the inner sinks of R1, R2, and R3, the laser beam was refocused at the newly etched surface of layer 1 by moving down the vertical *Z* axis by 35, 70, and 140 µm, respectively. 

### 3.5. Calibration Curves as Models to Predict FLMP Parameters

Here, the calibration curves produced in Section 3.2 and Section 3.3 were used as models to predict the processing parameters required to fabricate the 3D multi-depth reservoir micromodel. This afforded us the ability to test the accuracy of our model and the FLMP method. From the CAD in Figure 6, the reservoirs—R1, R2, and R3, have the same XY dimensions but different depths of 35, 70, and 140 µm for the reservoir matrices/outer sinks, and a total depth of 70, 140, and 280 µm for the inner circular sinks, respectively. The etch depth dependence on ${\mathrm{LF}}_{\mathrm{av}}$ calibration curve requires that all FLMP parameters be kept constant while varying ${\mathrm{LF}}_{\mathrm{av}}$ to achieve the required etch depth. Alternatively, the etch depth dependence on ${\mathrm{PS}}_{\mathrm{CNC}}$ calibration curve was used due to ease of control of ${\mathrm{PS}}_{\mathrm{CNC}}$ in comparison to ${\mathrm{LF}}_{\mathrm{av}}$ in our experimental setup. The predicted ${\mathrm{PS}}_{\mathrm{CNC}}$ necessary to achieve the desired etch depths across the reservoir micromodel are given in Table 2 and will be discussed later. 

### 3.6. Characterization of 3D Multi-Depth Reservoir Micromodel in Borosilicate Glass

**Figure 7:**
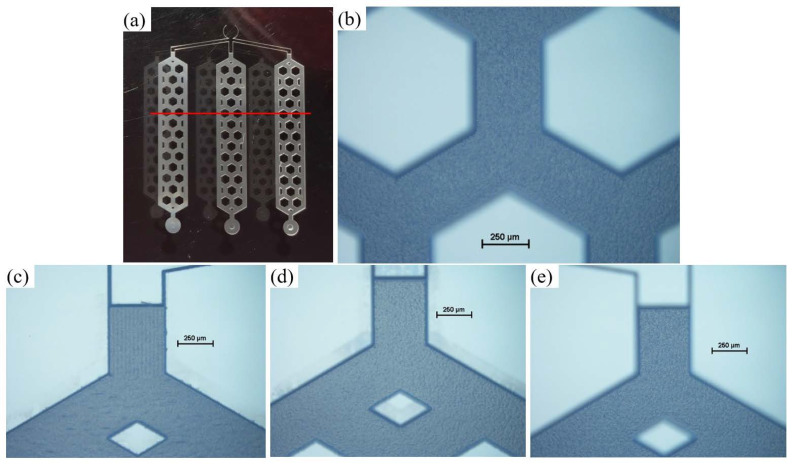
Images of several sections of the FLMP fabricated 3D multi-depth reservoir micromodel: (**a**) the red line illustrates the line profile scan path which goes through three pore spaces and two hexagonal pore bodies for each reservoir (R), (**b**) a zoom-in section of reservoir 3 where the image was focused at the etched surface, and (**c**–**e**) shows inlet portions of reservoirs 1, 2, and 3, respectively. Images (**b**,**e**) looks blurrier than (**c**,**d**) due to deeper depth as the microscope was focused on the bases of the channels. The scale bars are 250 µm.

Images of the 3D multi-depth reservoir micromodel machined in borosilicate glass using FLMP are shown in Figure 7. The overview of the micromodel (Figure 7a) covers approximately 20 mm × 15 mm surface. It took ~10 h to machine all the various components and depths of the micromodel. Figure 7b shows a portion of the R2 matrix that highlights the solid grains, i.e., hexagon and trapezoid geometries, that are separated from each other by a homogenous micro channel network. The red line indicates the surface profilometer path used to scan the etch depths for R1, R2, and R2 matrices. A continuous line profile scan across each reservoir, as shown by the red line, produces three depth measurements for each R that was expected to be equal. A similar approach was used to measure the etch depths across the circular sinks. Figure 7c–e show the respective portions of R1, R2, and R3 inlet channels. Here, it is shown that FLMP can make both sharp and curved etch features unlike wet photolithography methods that produce curved/rounded features as reported by others [13,66].

Through visual inspection, portions of the R1 inlet channel showed high surface roughness. The average surface roughness, *Ra*, was measured at multiple locations across the etched surface of the reservoirs and resulted in *Ra* values of 525, 320, and 800 nm for R1, R2, and R3, respectively. The *Ra* for the unmachined glass substrate was ~0.5 nm. Each value was obtained by averaging 5 experimental measurements. This *Ra* data set is not enough to predict a meaningful relationship, such as the dependence of *Ra* on etch depth and/or *Ra* on ${\mathrm{PS}}_{\mathrm{CNC}}$. Details of this comprehensive investigation will be presented in another manuscript as previously mentioned. However, it is worth mentioning that the highest *Ra* value of 800 nm obtained for R3, which has the deepest etch depth relative to R1 and R2, agrees with reports by others [48,52]. At constant ${\mathrm{LF}}_{\mathrm{av}}$, deeper channels (e.g., R3 with 140 µm etch depth) are obtained at slower ${\mathrm{PS}}_{\mathrm{CNC}}$ (0.142 mm/s) in comparison to etch depths of R2 (70 µm) or R1 (35 µm) that were machined at higher speeds of 0.485 and 1.301 mm/s, respectively. The slower ${\mathrm{PS}}_{\mathrm{CNC}}$ increases thermal effects due to proximity and overlap of laser pulses that is accompanied by debris build up. The debris occupies the channels, blocking the laser beam to the desired target surfaces which increases the surface roughness. The roughness of the unetched glass surfaces right next to the etched structures was also determined by collecting surface profilometer scans at multiple locations, including the inlet, matrix, and sinks of all 3 reservoirs. Each data point was recorded near the etched structures by a 100 µm long scan. More than 20 data points were averaged to produce an *Ra* of 3.7 nm with a wide deviation of ±5.1 nm. This range of roughness (3.7 ± 5.1 nm) around the etched features compares reasonably well to the furthest (>2000 µm) unetched area roughness of ~0.5 nm. This should not pose challenges for applications requiring bonding of a lid to the top of the borosilicate glass substrate to form a sealed channel or chamber.

**Figure 8:**
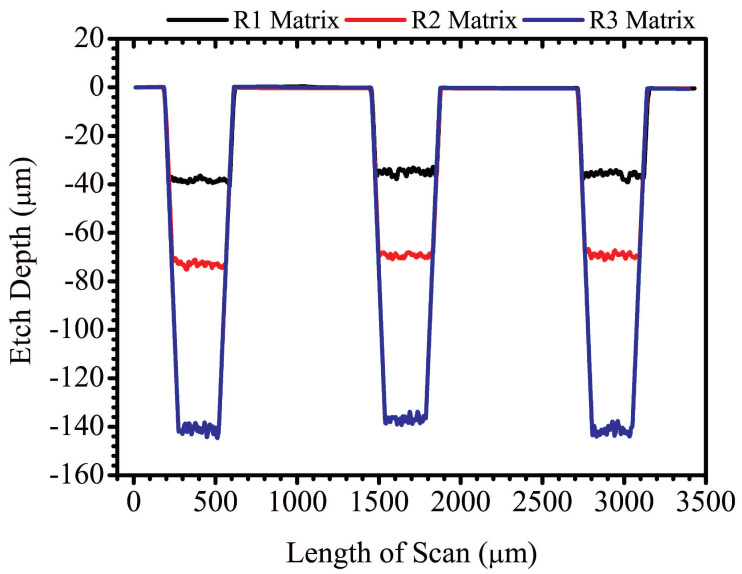
A plot showing the surface profilometer line scans across the matrices of the 3D multi-depth reservoir micromodel shown in Figure 7a. The black, red, and blue traces correspond to the profiles of reservoirs R1, R2, and R3, respectively.

**Figure 9:**
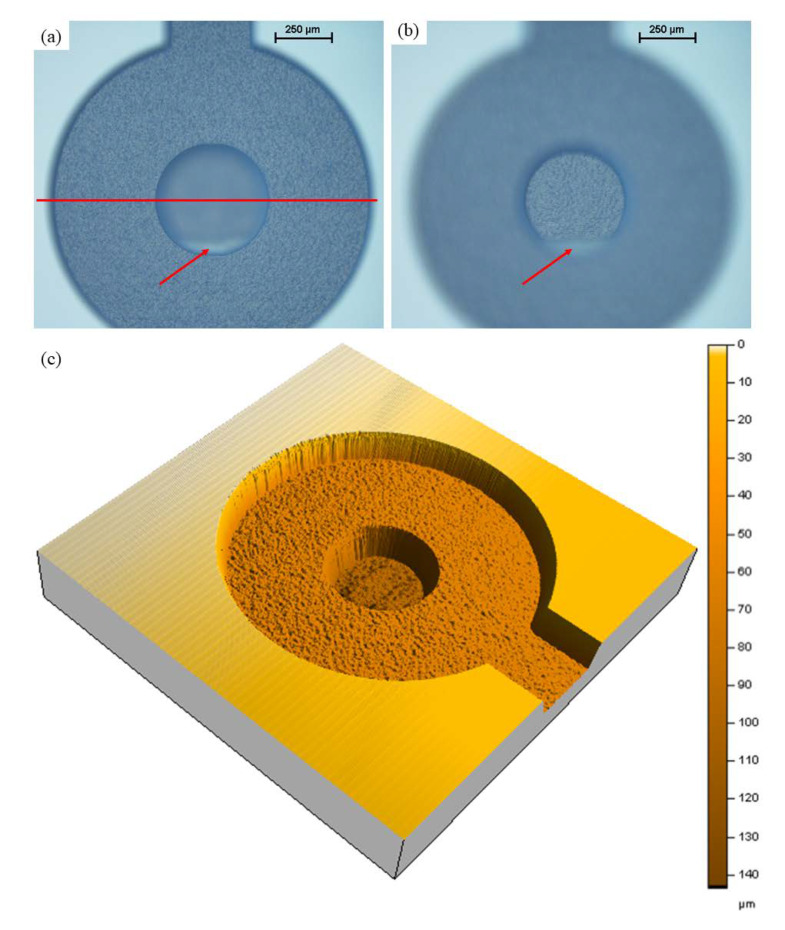
Images of the 3D multi-depth reservoir micromodel (**a**,**b**) taken with an optical microscope and showing the (**a**) outer and (**b**) inner circular sinks of R3 in focus. The red line across (**a**) indicates a 2D line profile scan path while (**c**) is a 3D image of reservoir R2 sink recorded with the surface profilometer at 2 mg force, 10 µm/s speed, 10 Hz, and 3 µm scan interval. The arrows in (**a**,**b**) point to the location of machined debris remaining after sonication.

Figure 8 shows a line profile scan across the reservoir matrices. The black, red, and blue traces represent the line profile scans across the matrices of R1, R2, and R3, respectively. The line profile scans are vertically stacked up in the graph, and this illustrates how neatly all the etch profiles overlap across the matrices of all three reservoirs. This agrees with the etch profile shown in Figure 3. Also, it shows the robustness of the FLMP technique which makes it possible to use calibration curves as models to predict experimental parameters required for future experiments. The measured etch depths, including standard deviations, and percentage errors are given in Table 2 above. The average etch depths for the matrices of R1, R2, and R3 were 36.3, 70.0, and 140.0 µm in comparison to the model prediction values of 35, 70.0, and 140.0 µm, which produces percentage errors of 3.7, 0.0, and 0.0%, respectively. This shows that our approach of using FLMP technique to machine 3D multi-depth features has good accuracy in producing the required results predicted by the calibration model. Also, the results in Table 2 shows standard deviations (σ) of ≤ 2 µm, indicating a good repeatability of etch depths across the large etch surface of all three reservoirs. Optical microscope and surface profilometer images of the sinks of R3 and R2 are shown in Figure 9. The images of Figure 9a and Figure 9b, were recorded consecutively by focusing the microscope at the etch surfaces of the outer and inner sinks of R3, respectively. Some remaining debris can be seen in both images at the lower portion of the inner sink; a common occurrence observed by other investigators in deep channels and pockets machined by FLMP [48,52,56,59]. This indicates that >30 min of sonication in isopropyl alcohol bath was probably required to remove all remaining debris. The 3D image of reservoir R2 sink in Figure 9c provides a complementary visual observation to the microscope images. It emphasizes the vertical depth information where the 2D microscope is lacking. The recorded total etch depth of 144 µm in Figure 9c differs marginally by ~6 µm in comparison to the combined 2D line profile etch depths of the outer and inner sinks of R2 (138 µm).

**Figure 10:**
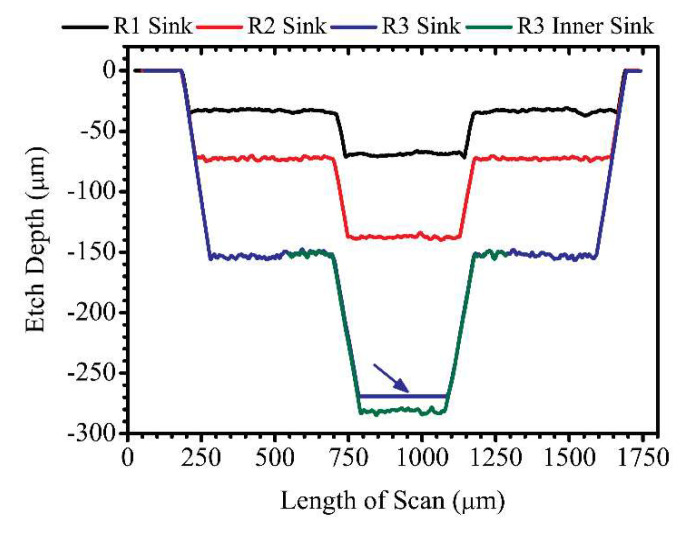
A plot of line profile scans across the circular sinks of the 3D multi-depth reservoir micromodel. The black, red, and blue traces correspond to the profiles of reservoirs R1, R2, and R3, respectively. The green trace was a repeated scan for reservoir R3 from the newly etched outer sink surface (layer 1) due to instrument limit which is observed as a smooth horizontal etch surface (indicated by blue arrow).

A graph showing 2D line profile scans across the sinks of R1, R2, and R3 is shown in Figure 10 and their measured etch depths are given in Table 2, showing the different etch depths. The instrument limit of the surface profilometer was ~270 µm which was observed by a linear and smooth horizontal etch surface (blue trace arrow) in R3. Therefore, another profile scan was done by starting from the etched layer 1 surface of R3 (green trace). Here also, a good overlap was observed between the two profiles (blue and green traces). 

The average experimental etch depth values obtained for the outer sinks of R1, R2, and R3 were 33.1 ± 0.1, 72.3 ± 0.3, and 152.2 ± 0.1 µm with percentage errors of −5.4, 3.3, and 8.7% in comparison to the calibration model prediction values of 35.0, 70.0, and 140.0 µm, respectively. The smaller deviations (σ ≤ 0.3 µm) observed for the outer sinks in comparison to the matrices (σ ≤ 2.0 µm) of the reservoirs could be due to localized etching in the former than the latter. For example, the experimental etch depth values of 33.0 and 33.2 µm for R1 outer sink were machined in a relatively small surface area while that of the matrix (38.4, 34.8, and 35.7 µm) was spread over a large etch area as shown in Figure 7 and Figure 9. This would allow small variations in the substrate, such as material density and surface height fluctuations, to slightly impact the resultant etch depths. 

A similar range of percentage errors was obtained for the inner sinks (3.1–7.9%) relative to the outer sinks (3.3–8.7%) as shown in Table 2. Here, experimental etch depth values of 36.1, 65.2, and 128.9 µm were obtained for the inner sinks of R1, R2, and R3 with respective percentage errors of 3.1, −6.9, and −7.9%. Generally, it was observed that etch depths in layer 2 (i.e., inner sinks) were shallower than the values predicted by the model while those in layer 1 (matrices and outer sinks) had deeper depths. The etch depths of R2 (65.2 µm) and R3 (128.9 µm) inner sinks were shallower than their respective model predicted values of 70.0 and 140.0 µm, except R1 inner sink (36.1 µm) which was deeper than the predicted value of 35.0 µm. On the contrary, most of the etch depths in layer 1—i.e., R1, R2 and R3 matrices, and R2 and R3 outer sinks, except R1 outer sink, were deeper than their respective values predicted by the calibration model as shown in Table 2. This was largely attributed to remaining debris on the surface of layer 1 that partially impedes the laser beam from direct interaction with the etched surface during the machining of layer 2 features as previously discussed above and reported in the literature by others [48,52,56,59]. The remaining debris on the layer 1 etched surface competes with material removal in layer 2, slightly impacting the efficiency of the etching process which results in reduction in predicted etch depths as observed here. Also, another reason for observing shallower etch depths in deeper channels is the limitation on etch volume due to the beams focal volume at constant Z position (focal distance)—i.e., the volume of material removed decreases significantly beyond the region where the fluence is tightly focused [39,44]. However, in Section 3.2, etch depths of 251.1 and 270.7 µm, which are deeper than the predicted values of R2 (70.0 µm) and R3 (140.0 µm) inner sinks, were successfully obtained due to their low aspect ratios (<0.2) relative to that of the inner sinks (<0.4). Therefore, the major factor responsible for the shallower than predicted etch depths for most of the layer 2 features is largely attributed to the impedance by the remaining debris on layer 1 to the laser beam.

## 4. Conclusions

The fabrication of a 4-depth 3D reservoir micromodel over a large surface area (20 mm × 15 mm) in a borosilicate glass substrate has been reported, for the first time, using femtosecond laser material processing (FLMP). The etch profile of the focused laser beam showed a Gaussian-like profile that makes ~8° contact angle with the vertical plane. The dependence of etch depth on two major FLMP parameters – average laser fluence (${\mathrm{LF}}_{\mathrm{av}}$), and CNC processing speed (${\mathrm{PS}}_{\mathrm{CNC}}$), was studied. It was found that etch depth has a strong linear dependence on ${\mathrm{LF}}_{\mathrm{av}}$ with an excellent $R^{2}$ value of 0.991. Also, a threshold average laser fluence (${\mathrm{LF}}_{\mathrm{av}}^{\mathrm{th}}$) value of 22.72 J/cm^2^ was determined as the minimum energy density required to etch borosilicate glass. The experimental data points for etch depth dependence on ${\mathrm{PS}}_{\mathrm{CNC}}$ was fitted to an inverse, logarithm, and exponential relations. Our data for borosilicate glass was in better agreement with the exponential relation, while other substrates such as silicon, have been shown in the literature to agree more with an inverse relation. It was shown that etch depth has a three-phase exponential decay dependence on ${\mathrm{PS}}_{\mathrm{CNC}}$, with another excellent $R^{2}$ value of 0.991. The linear and three-phase exponential decay relationships were successfully used as models to predict processing parameters required to machine the 3D multi-depth reservoir micromodel. 

The etch depth dependence on ${\mathrm{PS}}_{\mathrm{CNC}}$ model was used to machine the 3D reservoir micromodel composed of 4 etch depths, i.e., 35, 70, 140, and 280 µm, in the same borosilicate glass substrate. The experimental etch depths showed good results accuracy with percentage errors ≤ 8.7% in comparison to the model prediction values. Deviations of ≤ 2.0 µm in depth were achieved which showed that the etch depths were repeatable across the large etched surface of the 3-reservoir micromodel consisting of 4 multiple depths. Thus, this study has shown the robustness of FLMP as a fabrication technique to produce reliable etch depth results across a large surface area in a borosilicate glass substrate. In addition, it was shown that the etch depth dependency models produced in this study will be useful to guide the work of future researchers. This study will help the development and fabrication of micro/nanotechnology (MNT) systems, including microfluidic devices that are used for transport investigations in porous media. 

## Figures and Tables

**Table 1:** Processing parameters used for FLMP of borosilicate glass substrate.

Processing Parameters	Value	Unit
Pulse width (τ)	100	fs
Wavelength (λ)	800	nm
Repetition rate	1	kHz
Beam diameter	12.3	µm
Pitch (center-to-center) of beam	5	µm
CNC speed (${\mathrm{PS}}_{\mathrm{CNC}}$)	0.025–10	mm/s
Average laser fluence (${\mathrm{LF}}_{\mathrm{av}}$)	14.31–1388.62	J/cm^2^
Resultant etch depth	3.5–223.8	µm

**Table 2:** Surface profilometer depth characterization of the 3D multi-depth reservoir micromodel machined into borosilicate glass substrate using FLMP. The experimental machined etch depths are compared to model predictions. The depths of layer 2 are relative to the etched surface of layer 1. NB: (-) % error indicates that the machined etch depth value was < values predicted by the calibration model.

Reservoirs		CAD Etch Depth (µm)	Etch Depth vs. $PS_{CNC}$ Model Prediction (mm/s)	Experimental Etch Depth (µm)			Average ± σ (µm)	%Error
Layer 1	R1 Matrix	35.0	1.301	38.4	34.8	35.7	36.3 ± 1.9	3.7
	R2 Matrix	70.0	0.485	71.6	69.3	69.2	70.0 ± 1.4	0.0
	R3 Matrix	140.0	0.142	141.2	137.6	141.0	140.0 ± 2.0	0.0
	R1 Outer Sink	35.0	1.301	33.0	33.2	-	33.1 ± 0.1	−5.4
	R2 Outer Sink	70.0	0.485	72.5	72.1	-	72.3 ± 0.3	3.3
	R3 Outer Sink	140.0	0.142	152.3	152.1	-	152.2 ± 0.1	8.7
Layer 2	R1 Inner Sink	35.0	1.301	36.1	-	-	36.1	3.1
	R2 Inner Sink	70.0	0.485	65.2	-	-	65.2	−6.9
	R3 Inner Sink	140.0	0.142	128.9	-	-	128.9	−7.9

## References

[1] McKellar M., Wardlaw N.C. (1982). A Method of Making Two-Dimensional Glass Micromodels of Pore Systems. J. Can. Pet. Technol..

[2] Lago M., Huerta M., Gomes R. (2002). Visualization Study during Depletion Experiments of Venezuelan Heavy Oils Using Glass Micromodels. J. Can. Pet. Technol..

[3] Mejia L., Tagavifar M., Xu K., Mejia M., Du Y., Balhoff M. (2019). Surfactant Flooding in Oil-Wet Micromodels with High Permeability Fractures. Fuel.

[4] Lyu X., Liu H., Pang Z., Sun Z. (2018). Visualized Study of Thermochemistry Assisted Steam Flooding to Improve Oil Recovery in Heavy Oil Reservoir with Glass Micromodels. Fuel.

[5] Yun W., Chang S., Cogswell D.A., Eichmann S.L., Gizzatov A., Thomas G., Al-Hazza N., Abdel-Fattah A., Wang W. (2020). Toward Reservoir-on-a-Chip: Rapid Performance Evaluation of Enhanced Oil Recovery Surfactants for Carbonate Reservoirs Using A Calcite-Coated Micromodel. Sci. Rep..

[6] Chalbaud C., Robin M., Lombard J.-M., Bertin H., Egermann P. (2010). Brine/CO2 Interfacial Properties and Effects on CO2 Storage in Deep Saline Aquifers. Oil Gas Sci. Technol. Rev. d’IFP Energies Nouv..

[7] Morais S., Liu N., Diouf A., Bernard D., Lecoutre C., Garrabos Y., Marre S. (2016). Monitoring CO2 Invasion Processes at the Pore Scale Using Geological Labs on Chip. Lab Chip.

[8] Song W., de Haas T.W., Fadaei H., Sinton D. (2014). Chip-off-the-Old-Rock: The Study of Reservoir-Relevant Geological Processes with Real-Rock Micromodels. Lab Chip.

[9] Zhang J., Zhang H., Lee D., Ryu S., Kim S. (2020). Microfluidic Study on the Two-Phase Fluid Flow in Porous Media during Repetitive Drainage-Imbibition Cycles and Implications to the CAES Operation. Transp. Porous Media.

[10] Seyyedi M., Giwelli A., White C., Esteban L., Verrall M., Clennell B. (2020). Effects of Geochemical Reactions on Multi-Phase Flow in Porous Media during CO2 Injection. Fuel.

[11] Sinton D. (2014). Energy: The Microfluidic Frontier. Lab Chip.

[12] Tagavifar M., Xu K., Jang S.H., Balhoff M.T., Pope G.A. (2017). Spontaneous and Flow-Driven Interfacial Phase Change: Dynamics of Microemulsion Formation at the Pore Scale. Langmuir.

[13] Conn C.A., Ma K., Hirasaki G.J., Biswal S.L. (2014). Visualizing Oil Displacement with Foam in A Microfluidic Device with Permeability Contrast. Lab Chip.

[14] Rossen W.R. (2008). Comment on “Verification of Roof Snap-off as a Foam-Generation Mechanism in Porous Media at Steady State”. Colloids Surf. A Physicochem. Eng. Asp..

[15] Krummel A.T., Datta S.S., Münster S., Weitz D.A. (2013). Visualizing Multiphase Flow and Trapped Fluid Configurations in A Model Three-Dimensional Porous Medium. AIChE J..

[16] Xu K., Liang T., Zhu P., Qi P., Lu J., Huh C., Balhoff M. (2017). A 2.5-D Glass Micromodel for Investigation of Multi-Phase Flow in Porous Media. Lab Chip.

[17] Anbari A., Chien H.-T., Datta S.S., Deng W., Weitz D.A., Fan J. (2018). Microfluidic Model Porous Media: Fabrication and Applications. Small Nano Micro..

[18] Chrysikopoulos C.V., Plega C.C., Katzourakis V.E. (2011). Non-Invasive in Situ Concentration Determination of Fluorescent or Color Tracers and Pollutants in A Glass Pore Network Model. J. Hazard. Mater..

[19] Yun W., Ross C.M., Roman S., Kovscek A.R. (2017). Creation of A Dual-Porosity and Dual-Depth Micromodel for the Study of Multiphase Flow in Complex Porous Media. Lab Chip.

[20] Karadimitriou N.K., Joekar-Niasar V., Hassanizadeh S.M., Kleingeld P.J., Pyrak-Nolte L.J. (2012). A Novel Deep Reactive Ion Etched (DRIE) Glass Micro-Model for Two-Phase Flow Experiments. Lab Chip.

[21] Thienot E., Domingo F., Cambril E., Gosse C. (2006). Reactive Ion Etching of Glass for Biochip Applications: Composition Effects and Surface Damages. Microelectron. Eng..

[22] Wu B., Kumar A., Pamarthy S. (2010). High Aspect Ratio Silicon Etch: A Review. J. Appl. Phys..

[23] Karadimitriou N.K., Hassanizadeh S.M. (2012). A Review of Micromodels and Their Use in Two-Phase Flow Studies. Vadose Zo. J..

[24] Au A.K., Huynh W., Horowitz L.F., Folch A. (2016). 3D-Printed Microfluidics. Angew. Chem. Int. Ed..

[25] Seers T.D., Alyafei N. (2018). Open Source Toolkit for Micro-Model Generation Using 3D Printing. SPE Europec Featured at 80th EAGE Conference and Exhibition, Copenhagen, Denmark.

[26] Giridhar M.S., Seong K., Schülzgen A., Khulbe P., Peyghambarian N., Mansuripur M. (2004). Femtosecond Pulsed Laser Micromachining of Glass Substrates with Application to Microfluidic Devices. Appl. Opt..

[27] Sugioka K., Cheng Y., Midorikawa K. (2005). Three-Dimensional Micromachining of Glass Using Femtosecond Laser for Lab-on-a-Chip Device Manufacture. Appl. Phys. A.

[28] Hayden C.J., Dalton C. (2010). Direct Patterning of Microelectrode Arrays Using Femtosecond Laser Micromachining. Appl. Surf. Sci..

[29] Italia V., Giakoumaki A.N., Bonfadini S., Bharadwaj V., Le Phu T., Eaton S.M., Ramponi R., Bergamini G., Lanzani G., Criante L. (2018). Laser-Inscribed Glass Microfluidic Device for Non-Mixing Flow of Miscible Solvents. Micromachines.

[30] Fulton A.L., Beebe D.J., Sackmann E.K., Fulton A.L., Beebe D.J. (2014). The Present and Future Role of Microfluidics in Biomedical Research. Nature.

[31] Nguyen N.-T., Wu Z. (2005). Micromixers—A Review. J. Micromech. Microeng..

[32] Chang T.-C., Wang S.-C., Chien C.-W., Cheng C.-W., Lee C.-Y. (2011). Using Femtosecond Laser to Fabricate the Interior 3D Structures of Polymeric Microfluidic Biochips. J. Laser Micro Nanoeng. JLMN.

[33] Dalton C., Hayden C.J., Burt J.P., Manz A., Eijkel J.C.T., Burt J.P.H. (2003). A Circular Ac Magnetohydrodynamic Micropump for Chromatographic Applications. Sens. Actuators B Chem..

[34] Voldman J., Jaffe A. (2018). Multi-Frequency Dielectrophoretic Characterization of Single Cells. Microsyst. Nanoeng..

[35] Khalil A.A., Lalanne P., Bérubé J.-P., Petit Y., Vallée R., Canioni L. (2019). Femtosecond Laser Writing of Near-Surface Waveguides for Refractive-Index Sensing. Opt. Express.

[36] Balaji V., Castro K., Folch A., Balaji V., Castro K., Folch A. (2018). A Laser-Engraving Technique for Portable Micropneumatic Oscillators. Micromachines.

[37] Chen G.Y., Piantedosi F., Otten D., Kang Y.Q., Zhang W.Q., Zhou X., Monro T.M., Lancaster D.G. (2018). Femtosecond-Laser-Written Microstructured Waveguides in BK7 Glass. Sci. Rep..

[38] Owusu-Ansah E., Dalton C., Cully C. Femtosecond Laser Machining of Complex X-Ray Masks in Tungsten Sheets.

[39] Phillips K.C., Gandhi H.H., Mazur E., Sundaram S.K. (2015). Ultrafast Laser Processing of Materials: A Review. Adv. Opt. Photonics.

[40] De Angelis R., Duvillaret L., Andreoli P.L., Cipriani M., Consoli F., De Angelis R., Duvillaret L., Andreoli P.L., Cipriani M., Cristofari G. (2016). Time-Resolved Absolute Measurements by Electro-Optic Effect of Giant Electromagnetic Pulses Due to Laser-Plasma Interaction in Nanosecond Regime. Sci. Rep..

[41] Owusu-Ansah E., Horwood C.A., El-Sayed H.A., Birss V.I., Shi Y.J. (2015). A Method for the Formation of Pt Metal Nanoparticle Arrays Using Nanosecond Pulsed Laser Dewetting. Appl. Phys. Lett..

[42] Owusu-Ansah E., Birss V.I., Shi Y. (2020). Mechanisms of Pulsed Laser-Induced Dewetting of Thin Platinum Films on Tantalum Substrates—A Quantitative Study. J. Phys. Chem. C.

[43] Winkler M.T. (2009). Non-Equilbrium Chalcogen Concentrations in Silicon: Physical Structure, Electronic Transport, and Photovoltaic Potential. PhD Thesis.

[44] Sugioka K., Cheng Y. (2014). Femtosecond Laser Three-Dimensional Micro- and Nanofabrication. Appl. Phys. Rev..

[45] Garrido-Diez D., Baraia I. (2017). Review of Wide Bandgap Materials and Their Impact in New Power Devices. 2017 IEEE International Workshop of Electronics, Control, Measurement, Signals and their Application to Mechatronics (ECMSM).

[46] Ben-Yakar A., Byer R.L. (2004). Femtosecond Laser Ablation Properties of Borosilicate Glass. J. Appl. Phys..

[47] Hayden C.J. (2010). A Simple Three-Dimensional Computer Simulation Tool for Predicting Femtosecond Laser Micromachined Structures. J. Micromech. Microeng..

[48] Lee S., Yang D., Nikumb S. (2008). Femtosecond Laser Micromilling of Si Wafers. Appl. Surf. Sci..

[49] Li C., Nikumb S., Wong F. (2006). An Optimal Process of Femtosecond Laser Cutting of NiTi Shape Memory Alloy for Fabrication of Miniature Devices. Opt. Lasers Eng..

[50] Grojo D. (2019). Internal Structuring of Silicon by Ultrafast Laser Irradiation. 2019 Conference on Lasers and Electro-Optics Europe and European Quantum Electronics Conference.

[51] Stuart B.C., Feit M.D., Herman S., Rubenchik A.M., Shore B.W., Perry M.D. (1996). Optical Ablation by High-Power Short-Pulse Lasers. J. Opt. Soc. Am. B.

[52] Shah L., Mazumder J., Kam D.H., Shah L., Mazumder J. (2011). Femtosecond Laser Machining of Multi-Depth Microchannel Networks onto Silicon. J. Micromech. Microeng..

[53] Pfeiffer M., Engel A., Weissmantel S., Scholze S., Reisse G., Schmidt M., Zaeh M.F., Graf T., Ostendorf A. (2011). Microstructuring of Steel and Hard Metal Using Femtosecond Laser Pulses. Lasers in Manufacturing 2011: Proceedings of the Sixth International WLT Conference on Lasers in Manufacturing.

[54] Shin H., Kim H., Jang Y., Jung J., Oh J. (2017). Femtosecond Laser-Inscripted Direct Ultrafast Fabrication of A DNA Distributor Using Microfluidics. Appl. Sci..

[55] Lei C., Pan Z., Jianxiong C., Tu P. (2018). Influence of Processing Parameters on the Structure Size of Microchannel Processed by Femtosecond Laser. Opt. Laser Technol..

[56] Huang Y., Wu X., Liu H., Jiang H. (2017). Fabrication of Through-Wafer 3D Microfluidics in Silicon Carbide Using Femtosecond Laser. J. Micromech. Microeng..

[57] Ito Y., Yoshizaki R., Miyamoto N., Sugita N. (2018). Ultrafast and Precision Drilling of Glass by Selective Absorption of Fiber-Laser Pulse into Femtosecond-Laser-Induced Filament. Appl. Phys. Lett..

[58] Chalupský J., Krzywinski J., Juha L., Hájková V., Cihelka J., Burian T., Vyšín L., Gaudin J., Gleeson A., Jurek M. (2010). Spot Size Characterization of Focused Non-Gaussian X-Ray Laser Beams. Opt. Express.

[59] Crawford T.H.R., Borowiec A., Haugen H.K. (2005). Femtosecond Laser Micromachining of Grooves in Silicon with 800 Nm Pulses. Appl. Phys. A.

[60] Zamuruyev K.O., Zrodnikov Y., Davis C.E. (2016). Photolithography-Free Laser-Patterned {HF} Acid-Resistant Chromium-Polyimide Mask for Rapid Fabrication of Microfluidic Systems in Glass. J. Micromech. Microeng..

[61] Ameer-Beg S., Perrie W., Rathbone S., Wright J., Weaver W., Champoux H. (1998). Femtosecond Laser Microstructuring of Materials. Appl. Surf. Sci..

[62] Roth G.-L., Esen C., Hellmann R. (2017). Femtosecond Laser Direct Generation of 3D-Microfluidic Channels inside Bulk PMMA. Opt. Express.

[63] Khorshidian H., James L.A., Butt S.D. (2018). Demonstrating the Effect of Hydraulic Continuity of the Wetting Phase on the Performance of Pore Network Micromodels during Gas Assisted Gravity Drainage. J. Pet. Sci. Eng..

[64] Soleimani M. (2017). Naturally Fractured Hydrocarbon Reservoir Simulation by Elastic Fracture Modeling. Pet. Sci..

[65] Liu J., Xie H., Wang Q., Chen S., Hu Z. (2020). Influence of Pore Structure on Shale Gas Recovery with CO_2_ Sequestration: Insight Into Molecular Mechanisms. Energy Fuels.

[66] Auset M., Keller A.A. (2004). Pore-Scale Processes That Control Dispersion of Colloids in Saturated Porous Media. Water Resour. Res..

